# Design, production and immunomodulatory potency of a novel allergen bioparticle

**DOI:** 10.1371/journal.pone.0242867

**Published:** 2020-12-01

**Authors:** Véronique Gomord, Virginie Stordeur, Anne-Catherine Fitchette, Elizabeth D. Fixman, Guy Tropper, Lorna Garnier, Réjean Desgagnes, Sébastien Viel, Julie Couillard, Guillaume Beauverger, Sylvain Trepout, Brian J. Ward, Ronald van Ree, Loic Faye, Louis-P Vézina

**Affiliations:** 1 ANGANY Innovation, Val de Reuil, France; 2 ANGANY Inc, Québec, Québec, Canada; 3 McGill University Health Centre, Research Institute (RI MUHC), Montreal, Quebec, Canada; 4 Service d’Immunologie Biologique, Hospices Civils de Lyon, Hôpital Lyon Sud, Pierre-Bénite, France; 5 IR2 Inserm, Plateforme de microscopie électronique, INSERM US43/CNRS UMS2016, Institut Curie, Orsay, France; 6 Department of Experimental Immunology, Molecular and Translational Allergy, Amsterdam, Netherlands; University of Maryland School of Medicine, UNITED STATES

## Abstract

Allergen immunotherapy (AIT) is the only disease-modifying treatment with evidence for sustained efficacy. However, it is poorly developed compared to symptomatic drugs. The main reasons come from treatment duration implying monthly injections during 3 to 5 years or daily sublingual use, and the risk of allergic side-effects. To become a more attractive alternative to lifelong symptomatic drug use, improvements to AIT are needed. Among the most promising new immunotherapy strategies is the use of bioparticles for the presentation of target antigen to the immune system as they can elicit strong T cell and B cell immune responses. Virus-like particles (VLPs) are a specific class of bioparticles in which the structural and immunogenic constituents are from viral origin. However, VLPs are ill-suited for use in AIT as their antigenicity is linked to structure. Recently, synthetic biology has been used to produce artificial modular bioparticles, in which supramolecular assemblies are made of elements from heterogeneous biological sources promoting the design and use of in vivo-assembling enveloped bioparticles for viral and non-viral antigens presentation. We have used a coiled-coil hybrid assembly for the design of an enveloped bioparticle (eBP) that present trimers of the Der p 2 allergen at its surface, This bioparticle was produced as recombinant and in vivo assembled eBPs in plant. This allergen biotherapeutic was used to demonstrate i) the capacity of plants to produce synthetic supramolecular allergen bioparticles, and ii) the immunomodulatory potential of naturally-assembled allergen bioparticles. Our results show that allergens exposed on eBPs induced a very strong IgG response consisting predominantly of IgG2a in favor of the TH1 response. Finally, our results demonstrate that rDer p 2 present on the surface of BPs show a very limited potential to stimulate the basophil degranulation of patient allergic to this allergen which is predictive of a high safety potential.

## Introduction

Despite large increases in the incidence of allergic diseases such as hay fever, allergic asthma and food allergy over the last decades [1, 2], there have been few changes in the management of these conditions [3]. Although new biologic agents that target allergy-associated immune mediators show promise, they are expensive and provide only symptomatic relief rather than correcting the underlying abnormal immune response. As a result, symptoms typically re-occur upon cessation of treatment. Allergen immunotherapy (AIT)—commonly called desensitization—is the only disease-modifying treatment with evidence for sustained efficacy [4]. AIT has been used for over 100 years now, first only as subcutaneous immunotherapy (SCIT), and more recently also as drops or tablets for sublingual immunotherapy (SLIT). In both cases, the therapy is based on delivery of crude allergen extracts. For SCIT, European allergen extracts are usually formulated as depot preparations with e.g. aluminum hydroxide, aluminum phosphate or tyrosine, functioning as adjuvants. Depot preparations are less commonly used for bee and wasp venom AIT and in the USA, non-adjuvanted, aqueous allergen extracts are typically used for AIT. The available SLIT products are also adjuvant-free. Several recent systematic reviews have concluded that both SCIT and SLIT can be effective, perhaps with a slight superiority of SCIT [5–7] Despite the fact that AIT is the only disease-modifying treatment for allergies, it has only achieved a ‘niche position’ compared to symptomatic drugs. The main reasons for the limited clinical penetration of AIT are the burden of prolonged treatment (eg: 3 to 5 years of monthly injections or daily sublingual use), and the risk of allergic side-effects. More recently, the desirability of years of administration of aluminum-containing depot preparations, in particular in children, has been questioned in Europe [8]. To become a more attractive alternative to lifelong symptomatic drug use, improvements to AIT are needed that shorten treatment protocols, improve safety and offer alternatives to aluminum-containing depots.

Since the early 1990s, there have been many efforts to replace allergen extracts by recombinant versions of major allergens [3, 4]. Obviously, purified recombinant proteins would greatly simplify standardization and allow more accurate dosing. This approach was clinically tested for grass pollen and birch pollen allergy. In Phase II trials, efficacy and safety were demonstrated, but results were not clearly superior to already existing extract-based therapies. To justify further investments into recombinant allergen-based approaches, a clear added-value over existing therapies is needed: fewer injections, shorter protocols and increased safety. Simply replacing extracts by purified proteins will not achieve this. Several novel strategies to increase the efficacy and safety of AIT have been proposed such as hypoallergenic recombinant mutants of major allergens [9]; synthetic peptides representing T-cell epitopes [10, 11]; B-cell epitopes conjugated to immunogenic carrier proteins; larger overlapping peptides, engaging innate responses [12–14]; and alternative routes of administration, such as intra-nodal injection [15–17]. Some successful Phase II trials have been carried out, but none of these candidate products has successfully reached market authorization, either because they failed in Phase III or because proof for shorter and safer treatment protocols was not convincingly demonstrated [4]. New approaches that can achieve this leap forward for AIT are urgently needed.

Among the most promising new immunotherapy strategies is the use bioparticles for the presentation of target antigen to the immune system. There is now accumulating evidence that delivery of antigens in the form of bioparticles has a profound impact on the skewing of the immune response [18–20]. Most bioparticles contain repetitive displays of conformational epitopes that can elicit strong T cell and B cell responses, effectively making them high potency immunization ‘devices’ [21].

These advantages have clearly been realized with highly successful nanoparticle-based vaccines against human papilloma virus (HPV) and hepatitis B virus (HBV). Both vaccines are considered to be virus-like particles (VLPs), a specific class of bioparticles in which the structural and immunogenic constituents are of viral origin. The former (HPV-VLPs) results from self-assembly of recombinant HPV capsid L1 proteins. The latter (HBV-VLPs) is an example of a so-called ‘enveloped’ VLP (eVLP) in which a target antigen—in this case the HBV surface antigen—is embedded in the envelope of the host cell in which this antigen is expressed. This latter type of VLP is more complex but more flexible than the ‘capsid-type’ VLP since there is no requirement for self-assembly. However, assembly and budding of eVLPs require oligomerization and palmitoylation of the structural transmembrane domains of surface proteins in the ER [22–24]. In most virus-derived bioparticles, this oligomerization is carried out by one key domain of surface protein monomers, the stalk, through disulfide bonding, which results in oligomerization of the protein, including its transmembrane section.

Enveloped VLPs have an additional potential benefit, compared to capsid VLPs, for the presentation of antigens. It appears that membrane components of the host cell in which these VLPs are produced (eg: mammalian, insect, plant) may have immunostimulatory effects. Although the mechanisms of action have not yet been clearly defined, it has been shown repeatedly that the immunostimulatory effect of eVLPs does not require the addition of adjuvants, and eVLPs are said to be self-adjuvanted [25].

While attractive because of their known safety profile, existing VLP vaccines (capsid or enveloped) are poorly suited for use as possible ‘vectors’ for in AIT. In both types, antigenicity is linked to structure, i.e. their antigenic determinants are the same as their structural determinants. VLPs are supramolecular structures that will not assemble if their key core surface elements are not present, and as these elements bear major immunogenic determinants, these VLP vaccines will inevitably elicit an anti-viral immune response if they are used to present allergens. This interfering response is likely to be exacerbated in enveloped VLPs where the lipid components act as adjuvant. Ideally, AIT bioparticles would be designed to elicit a specific neutralizing immune response against a naturally-occurring allergen alone. For this to happen safely, AIT bioparticles cannot carry immunogenic determinants other than those of their target antigen and thus the principles of their design, and their make-up, differ substantially from that of the existing VLP vaccines.

Recently, synthetic biology has been used to produce artificial modular bioparticles, in which supra-molecular assemblies are made of elements from heterogeneous biological sources. This is a new approach to the design of antigen presentation devices.

This approach has been used to link small immunogenic peptides to self-assembling viral and bacteriophage capsid components [26, 27]. One such application is the use of a coiled-coil paired to an eVLP backbone for the presentation of an influenza virus membrane channeling protein [28–30]. In this hybrid influenza VLP, a coiled-coil derived from saccharomyces GCN4 was fused at the C-terminus to the transmembrane domain and the cytosolic tail (TM/CT) of hemagglutinin and to the ectodomain of channel protein M2 (M2e) at the N-terminus. This fusion was used both for oligomerization of the TM/CT domain and presentation of influenza protein M2e at its surface. In addition, this hybrid/modular VLP self-assembled *in vivo* as recombinant (and thus a true bioparticle), which demonstrated that GCN4 and possibly other coiled-coils are sufficient to ensure oligomerization of the TM/CT domains of membrane proteins and subsequent budding of the hybrid particle, independently of the presence of the distal domains of the corresponding surface protein. This opened the way to the design and use of hybrid, in vivo self-assembling enveloped bioparticles for the presentation of non-viral antigens. It also opened the way to the design and production of bioparticles that have no viral immunogenic determinants on their surface.

Presentation of allergens as surface proteins of *in vivo* self-assembled bioparticles has not been achieved. It represents a significant challenge and relies on the development of hybrid nano-bioparticles. We have used the coiled-coil hybrid assembly described by Compans and collaborators [30] as a model for the design of an enveloped bioparticle (eBP) that presents trimers of the Der p 2 allergen at its surface, and that is free of any surface viral antigenic determinants. These were produced as recombinant and *in vivo* assembled eBPs in *Nicotiana benthamiana*. This hybrid bioparticle, a candidate allergen biotherapeutic, was used to demonstrate i) the capacity of plants to produce synthetic supramolecular allergen bioparticles, and ii) the immunomodulatory potential of naturally-assembled synthetic allergen bioparticles.

Der p 2 was chosen as a model allergen for this study as its biological activity [31–40] and epidemiology [41–46] has been well characterized. Der p 2 is also one of the most potent and frequent house dust mite (HDM) allergens and is recognized by at least 80% of HDM-allergic patients.

## Materials and methods

### Animals

Female Balb/c mice (7-week old) were purchased from Charles River Laboratories (Montréal, QC, Canada). Animal studies were approved by the McGill University Animal Care Committee and performed following the Canadian Council on Animal Care guidelines.

### Molecular design and cDNA assemblies

**Fig 1:**
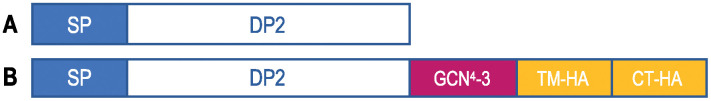
Schematic representation DNA encoding fusion protein used in this study. For construct aimed at producing supramolecular assemblies, a TM-HA/CT-HA domain from influenza hemagglutinin H5N1 (accession number: ABW06108.1), was fused at the C-terminus of the Der p 2 allergen (DP2).

For the expression of soluble recombinant Der p 2 (rDer p 2), the cDNA encoding the mature Der p 2 (accession number: AAF86462) was fused at the C-terminus to the tobacco chitinase signal sequence (Accession number: QEQ12695) and a cDNA encoding a hexa-histidine TAG was fused at the C-terminal end of the protein. For the expression of the Der p 2 hybrid supramolecular structure (BP-Der p 2), the cDNA encoding the coiled-coil yeast transcription factor GCN4 (accession number: 1GCM_A); [47] was fused at the N-terminus to the cDNA encoding the TM/CT domain of influenza virus hemagglutinin [30], and at the C-terminus, to the cDNA encoding the mature form of the Der p 2 allergen. Thus, the resulting construct was devoid of surface viral immunogenic determinants. A detailed illustration of the cDNA construct is shown in Fig 1. The sequences of all cDNAs described above were optimized for expression in *Nicotiana benthamiana*.

### Preparation of plasmids

Xba I / Kpn I and Sal I / Sac I restriction sites were respectively cloned at the 5' and 3' ends of each of the cDNA assemblies described above. These sites were then used to clone the cDNA assembly into the binary expression vector pAG01 (unpublished data, Gomord 2018) as described by Pagny and collaborators [48]. Vector pAG01 also contained an expression cassette for the silencing inhibitor p19 as described by Voinnet and collaborators [49, 50]. The vectors were then used to transform *Agrobacterium tumefaciens* strain LBA4404 and prepare an inoculum as described in [48].

### Production of Der p 2-specific antiserum

**Fig 2:**
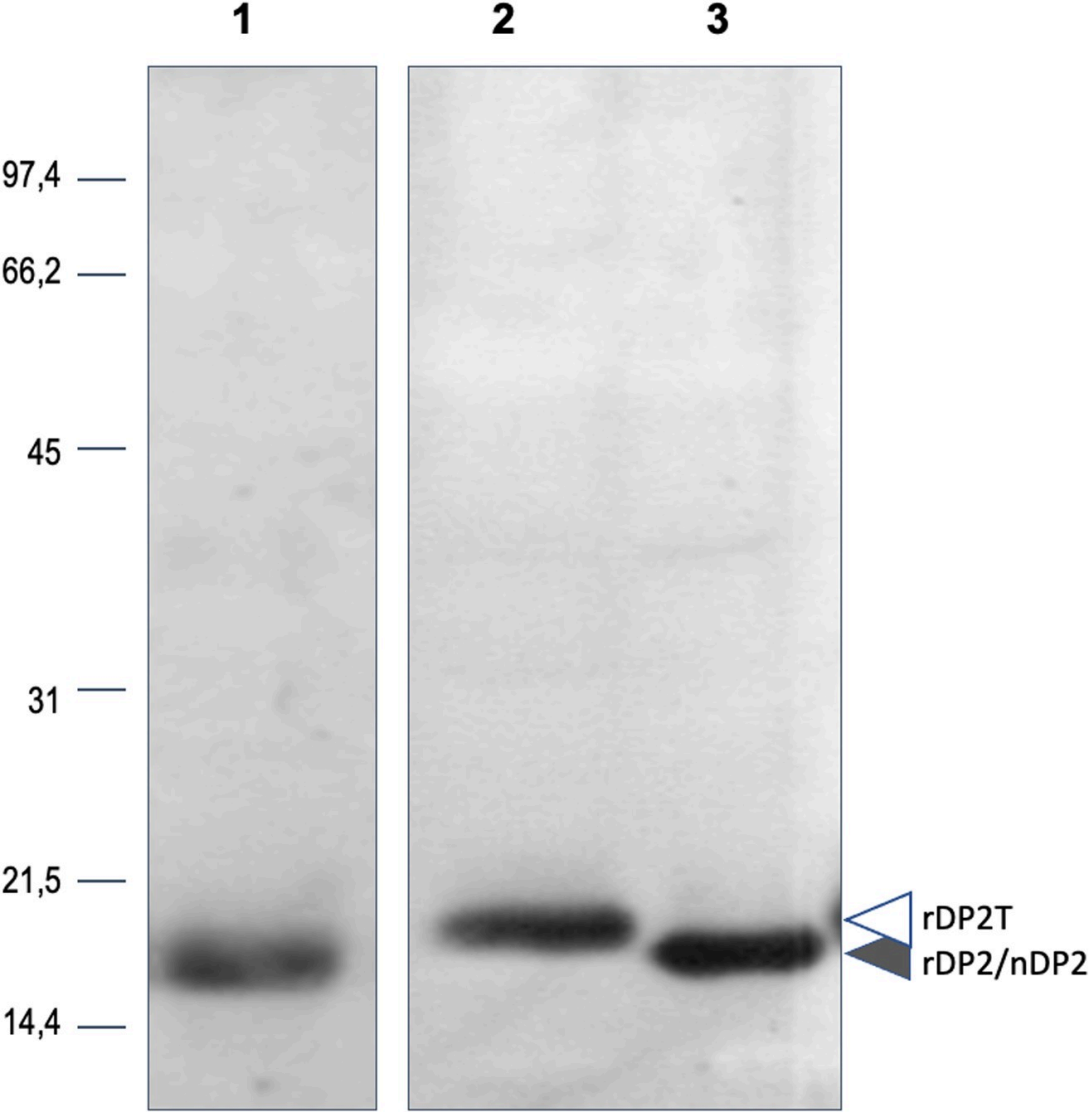
Characterization of Derp 2 immunserum. Der p 2 allergen from house dust mite (lane 1) or recombinant Der p 2 produced in plant with (lane 2) or without His tag (lane 3) were separated by SDS-PAGE and immunodetected with and immunodetected with anti-DP2 specific immunserum.

A partially-purified extract from *Dermatophagoides pteronyssinus* (Stallergenes # 06315L30) was injected intramuscularly in two rabbits. The immunization schedule of the rabbits was as described in [51]. Briefly, the partially purified extract from *D*. *pteronyssinus* was first dissolved in PBS at a concentration of 0.2mg/mL (allergen solution). Each injection consisted of 250uL of the allergen solution mixed with the same volume of Freund’s incomplete adjuvant except for the first injection, in which Freund’s complete adjuvant was used. The sera with the highest anti-nDer p 2 titre was then collected (Fig 2).

### Plant cultivation and transient expression

*N*. *benthamiana* seeds were sown in coco fiber plugs. The seedlings were grown for 14 days in a hydroponic system under continuous LED lighting and then transferred to larger hydroponic tanks containing a nutrient medium under LED lighting at 26° C and a 16hr/8hr day-night regimen where they were allowed to develop for 14 additional days. At the end of this period, their aerial part was immersed in a suspension of agrobacteria carrying the binary vector (the inoculum). The inoculum was then infiltrated in leaves by two cycles of vacuum (-0.8 Bar) /release. Following infiltration, plants were transferred to new hydroponic tanks for 4–6 days. Transfection of the T-DNA assembly to leaf cells, followed by transcription and translation then occurred during the 4–6 following days.

### Extraction and purification of soluble rDer p 2

Extracts were prepared by passage of the leaves of *N*. *benthamiana* through a domestic juicer (Angelia 5500) and diluted in 1V of phosphate buffer (50 mM Na-phosphate, pH 7.5 supplemented with NaCl (0.5 M)). After rapid filtration on miracloth 60 μm and clarification on Supracap K100 Depth filter capsule (Pall), the extracts were loaded on a nickel-affinity matrix (GE Healthcare Bio-Science Corporation, His Trap excel GE, ref: 17-3712-05). The Ni-affinity matrix was washed and the recombinant allergen was eluted with 0.2M imidazole in phosphate buffer. Fractions eluted from different chromatographic runs were pooled and concentrated on Amicon^®^ Ultracel^®^-3K (Millipore–Ref: UFC900324). Size-exclusion chromatography was performed on HiPrep^™^ 16/60—Sephacryl^™^ S-100 HR (GE Healthcare Bio-Science Corporation, ref: 17-1165-01) in a PBS buffer (50 mM Na phosphate, pH 7.5, containing 0.15M NaCl). Size-exclusion chromatography fractions containing rDer P 2 were subsequently pooled and concentrated on Amicon^®^ Ultracel^®^-3K (Millipore–Ref: UFC900324) for further use.

### Extraction and purification of Der p 2 bioparticles (BP-Der p 2)

Extracts were prepared by passage of the leaves of *N*. *benthamiana* through a domestic juicer (Angelia 5500). After rapid filtration on miracloth 60 m and centrifugation, the extracts were clarified by passage through ceramic UF membranes (T300, Tami Industries, Nyons France) against 12 volumes of PBS buffer (50 mM Na phosphate, pH 7.5, containing 0.15M NaCl). The same system was then used to concentrate the clarified bioparticle suspensions prior to size exclusion chromatography (Sephacryl S500HR, GE Healthcare Bio-Science Corporation) as described by D’Aoust and collaborators [52]. The elution fractions were monitored for their total protein content at 280 nm and their BP-Der p 2 content by western blotting. The relevant fractions were pooled, sterilized on 0.45μm filters and concentrated by centrifugation.

### SDS-PAGE and western blot analysis

For soluble allergens, samples were heated at 90° C for 5 min in denaturation buffer A (Tris 62.5 mM pH 6.8, 10% glycerol, 1% SDS and 2% β-mercaptoethanol). For bioparticles, samples were heated at 90° C for 5 min in denaturation buffer B (Tris 62.5 mM pH 6.8, 10% glycerol, 2.5% SDS and 5% β-mercaptoethanol). Both preparations were centrifuged at 8000 g for 15 min before loading on gels. SDS-PAGE was performed on 18% polyacrylamide gels. Following electrophoretic separation, gels were either silver-stained or transferred onto a nitrocellulose membrane (Amersham^™^ Protran^™^ 0.45μm NC) for immunodetection. The primary antibody used for immunodetection was a polyclonal rabbit-antisera directed against nDer p 2 at a 1:5,000 dilution followed by a secondary goat anti-rabbit IgG antibody coupled to horseradish peroxidase (Bio-Rad, Hercules, CA, USA) at a 1: 30,000- dilution. Western blots were visualized with Amersham^™^ ECL^™^ Western Blotting Detection Reagents.

### N-terminal amino acid sequencing

The N-terminal amino acid sequence of the purified proteins was determined with an Applied Biosystems 492 automated protein sequencer (Applied Biosystems, Foster City, CA, USA).

### Far UV circular dichroism

A structural analysis of rDer p 2 was performed by circular dichroism (CD) and compared to that of purified natural Der p 2 (Indoor biotech: NA-Der p 2–1). CD spectra were recorded on a Chirascan Plus spectrometer (Applied Photophysics, Ltd, UK) at a 50 μM protein concentration. Flat quartz cells with 0.1 mm pathlength filled with 30 μL of sample were used for analysis. The final result shown is the average of 5 independent runs.

### Peptide mass finger printing

Two μg of purified proteins were dissolved in 50mM ammonium bicarbonate containing 0.1% ProteaseMAX^™^ surfactant (Sigma) and 20 mM DTT, alkylated in 33 mM iodoacetamide, and then digested with 0.1 μg trypsin for 3 hours at 37°C. The resulting peptides were analyzed by nanoLC-MS/MS using an Impact HD ESI-QqTOF (Bruker) coupled to an Ultimate 3000 RSLC (Thermo Fisher) equipped with a C18 column (Acclaim^®^PepMap RSLC 75 μm ID, 25 cm, 5 μm particles and pore size at 100Å (Thermo Scientific)). Precursor mass and fragment mass were searched with initial and mass tolerance of 10 ppm and 0.05 Da, respectively. The search included fixed modification of carbamidomethyl cysteine. Data were analyzed using the PEAKS 7 software.

### Cryo-electron microscopy sample preparation

After purification, a 5 μl drop of the sample was subsequently deposited on a glow-discharged lacey 200 mesh holey carbon copper electron microscopy grid (Ted Pella). The grids were manually blotted using Whatman filter paper and plunge-frozen into liquid ethane at -174°C using a Leica EM-CPC equipment (Leica, Wetzlar, Germany). After freezing, the grids were stored in a liquid nitrogen tank until observation at the electron microscope.

### Cryo-electron microscopy observation

Frozen electron microscopy grids were mounted on a Gatan 914 high-tilt cryo-holder (Gatan, Pleasanton, CA, USA). Cryo-EM images were collected on JEOL 2200FS 200kV field emission gun electron microscope (JEOL, Tokyo, Japan). 2k by 2k images were collected using a Gatan US1000 slow-scan CCD camera. Energy-filtered (20 eV slit) images were collected at pixel sizes between 0.54 and 0.72 angstrom at the specimen level with nominal defocus between 5 and 3 μm depending on the experiment.

### Bio-particle size analysis (BP-Der p 2) by Tunable Resistive pulse sensing (TRSP)

All measurements were conducted using the qNano (Izon Science Ltd., NZ) and Izon Control Suite v.3.3. All samples were 200 times diluted in filtered, degassed PBS. For all qNano measurements, the lower and upper fluid cells contained 75μl of electrolyte buffer (filtered, degassed PBS), whilst the upper fluid cell contained 35 μl of sample. A detailed description of qNano can be found in [53] and [54]. After completing the preparation, particle concentration and diameter were measured with TRPS for at least 500 particles/sample.

### Murine allergen sensitization and challenge model

Mice were sensitized intraperitoneally on days 0 and 14 with or without 200μl of a 12.5% solution of Imject alum (Fisher Scientific, Edmonton, AB, Canada) in saline, either alone or with 1μg of rDer p 2 or the equivalent amount of Der p 2 delivered as BP-Der p 2 or present in a commercial house dust mite (HDM) extract (Stallergenes Greer, Lenoir, NC, USA). The quantity of Der p 2 in BP-Der p 2 and in the HDM extract was quantified by western blot. In HDM extract, 1μg Der p 2 was present in 41μg total HDM protein. Starting on day 21, all mice were lightly anaesthetized with isoflurane and challenged intranasally with 41μg total HDM protein in a 30μl volume on four consecutive days. After 24 hours, airway hyperresponsiveness (AHR) was assessed (see below), and bronchoalveolar lavage (BAL) fluid and blood were harvested for ex vivo analysis.

### Assessment of AHR and BAL fluid inflammation

Airway hyperreactivity (AHR) outcomes (resistance and elastance) were assessed using the flexiVent small animal ventilator (SCIREQ, Inc., Montréal, QC, Canada). Prior to measurements, mice were sedated with xylazine hydrochloride and anaesthetized with sodium pentobarbital. They were then tracheotomized, connected to the ventilator via a cannula, ventilated and subsequently paralyzed with pancuronium bromide. After an equilibration period, baseline measurements were taken. Then, methacholine (acetyl-β-methylcholine chloride, Sigma-Aldrich Canada Ltd., Oakville, ON, Canada) was delivered as an aerosol, using concentrations ranging from 3.125 to 50 mg/ml. Respiratory system resistance and elastance were recorded following each dose of methacholine and the highest value was kept for analysis, subject to a coefficient of determination ≥0.9. Afterward, bronchoalveolar lavage (BAL) was performed. Two separate lavages of sterile saline (1ml) were instilled into the lungs via the tracheal cannula and the lung cells combined following centrifugation. After quantifying total cell recovery, 8 x 10^4^ cells were centrifuged onto glass slides for staining with Hema 3^™^ stain set (Fisher Scientific, Kalamazoo, MI, USA). Differential cell counts were based on counts from a minimum of 300 cells per slide.

### Blood collection and serum antibody levels

After BAL, blood was collected by cardiac puncture. After 30 minutes at room temperature to facilitate coagulation, samples were centrifuged to collect serum, which was stored at -80C until used. For determination of total Der p 2-specific IgG levels, U-bottom high-binding 96-well ELISA plates (Greiner Bio-one, Frickenhausen, Germany) were coated with rDer p 2 (1 μg/ml) in 100 mM bicarbonate/carbonate buffer at pH 9.5 (50 μl/well, overnight at 4°C). For the standard curve, rDer p 2 was replaced by mouse IgG antibodies (Sigma, St. Louis, MO, USA) and wells were coated as above. Before and after each of the following steps, wells were washed with phosphate buffered saline (PBS). Wells were blocked for 60 minutes at 37°C with blocking buffer comprised of 2% bovine serum albumin (BSA; Sigma) in PBS-Tween 20 (0.05%; Fisher Scientific, Ottawa, ON, Canada) (150 μl/well). Serum samples were diluted 1:50 in blocking buffer and added to triplicate wells (50 μl/well) and incubated for 60 minutes at 37°C. Blocking buffer alone was added to standard curve wells. After washing, HRP-conjugated anti-mouse total IgG antibodies (Jackson ImmunoResearch Laboratories Inc., West Grove, PA, USA) diluted 1:10,000 in blocking buffer were added (75 μl/well, 60 minutes at 37°C). Plates were detected with 3,3’,5,5’-tetramethyl benzidine (TMB) substrate (Millipore, Billerica, MA, USA) and stopped after 15 min with 0.5M H_2_SO_4_. Plates were read at 450nm on an EL800 microplate reader (BioTek Instruments Inc., Winooski, VT, USA). The concentration of Der p 2-specific IgG antibodies was determined using the standard curve included on each plate.

Der p 2-specific mouse IgG_1_ and IgG_2a_ antibodies were measured by endpoint titers starting from a 1/50 serum dilution and using 2-fold dilutions. Coating, blocking, serum, secondary antibody and detection steps were as described above. Anti-IgG_1_-HRP and anti-IgG_2a_-HRP secondary antibodies were diluted 1:20,000 and 1:10,000 respectively (SouthernBiotech, Birmingham, AL, USA). The cut-off optical density (OD) reading was calculated by averaging 8 blank wells and multiplying by 3. The endpoint titer was related to the last OD value above the cut-off.

### Human basophil activation test (BAT)

Basophil activation test was performed using the Allergenicity kit^®^ (Beckman Coulter, France), according to manufacturer’s recommendations. For each patient, 6 concentrations of rDer p 2 and BP-Der p 2 were tested: 1000, 500, 100, 10 and 1ng/mL and 3 concentrations of HDM extract: 100, 1 and 0.01 ng/mL. Der p 2 sensitized or non-sensitized patients were selected on the basis of their reactivity to rDer p 2 from *E*. *coli* in an immunocap assay. Venous blood was collected in EDTA coated tubes. Blood samples were incubated with the rDer p 2, BP-Der p 2, or HDM extract dilutions, an activating solution containing Ca^2+^, heparin and fluorescent antibodies (CRTh2-FITC, CD3-PC5 and CD203c-PE) for 15 minutes in the dark. Samples were then fixed, lysed and analyzed on a Navios (Beckman Coulter, France) flow cytometer.

## Results

### Production, purification and characterization of rDer p 2

**Fig 3:**
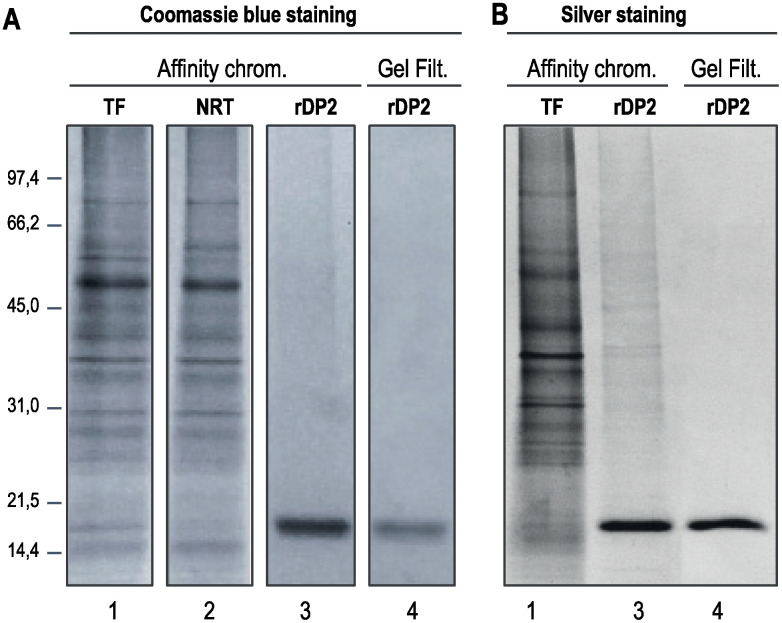
Purification of recombinant Der p 2 (rDP2T). Total proteins from clarified extract expressing rDP2 (lane 1), non-retained protein fraction (lane 2), retained protein fraction after nickel affinity chromatography (lane 3) and purified rDP2 obtained after size exclusion chromatography of the retained protein fraction (lane 4) were analysed by SDS-PAGE and protein Coomassie staining (A) or silver staining (B).

**Fig 4:**
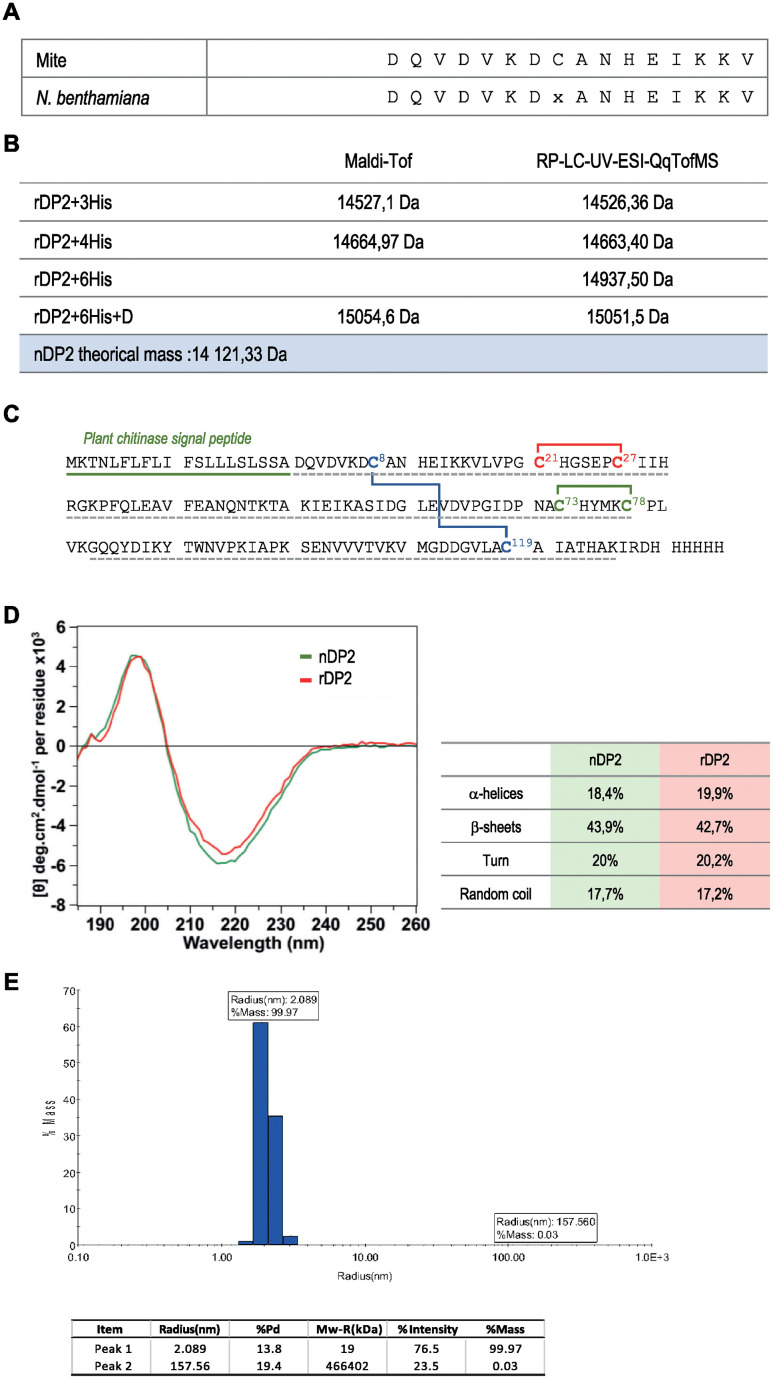
Post-translational modification of naturel (nDP2) and plant made Der p 2 allergen (rDP2). The physicochemical properties of the natural nDer p 2 and plant-made rDer p 2-His were compared by N-terminal sequencing (A), Mass measurement of intact rDP2 (B), peptide mapping mass spectrometry and disulfide bond analysis (C), and circular dichroism (D) after purification. Superimposed CD spectra of rDP2 and nDP2, samples are at 50 μM concentration, CD spectral range 185–260 nm, temperature: 20°C, (E) Characterization of DP2 using dynamic light scattering (step size: 1nm, bandwidth: 1nm).

rDer p 2 accumulated in *N*. *benthamiana* leaves during the 5 days following transfection. It was purified to near homogeneity from clarified whole leaf extracts by Ni-affinity chromatography followed by size-exclusion chromatography (Fig 3). Characterization by a combination of biochemical techniques demonstrated that rDer p 2 produced in *N*. *benthamiana* was indistinguishable from purified natural (n) Der p2) (Fig 4). In particular, MALDI-TOF analysis showed the presence of three disulfide bridges connecting cysteines 8–119, 21–27 and 73–78 (considering the mature Der p 2, sequence without peptide signal), as seen in the natural allergen from *Dermatophagoides* (Fig 4C). The molecular mass of purified rDer p 2 was found to be 14 937 Da by MALDI-TOF mass spectrometry, which is consistent with the theoretical molecular weight of nDer p 2, with the addition of the hexa-histidine residues of the tag (Fig 4B).

Circular dichroism demonstrated that the recombinant allergen expressed in *N*. *benthamiana leaves* was correctly folded (Fig 4D). More precisely, our data indicated that plant-produced rDer p2, as the purified nDer p 2, was mainly composed of beta sheets (Fig 4D) as described by Derewenda et al., [55]. Dynamic light scattering showed that the plant-produced rDer p 2 was present as a monomer, with barely detectable traces of polymeric and/or aggregated forms (Fig 4E).

Peptide mapping with a sequence coverage of 96% confirmed the amino acid sequence of rDer p 2. The identification of four peptides, all starting with the sequence DQVDVKD at their N-terminus, confirmed by the N-terminal sequencing, indicated that the signal peptide of pro-Der p 2 was cleaved at the same position and with the same efficacy in tobacco cells as in dust mite cells (Fig 4A).

Altogether, these data demonstrate that rDer p 2 transiently expressed in *N*. *benthamiana* has physico-chemical properties indistinguishable from those of its natural counterpart from HDM.

### Assembly of the hybrid construct in enveloped nanoparticles

**Fig 5:**
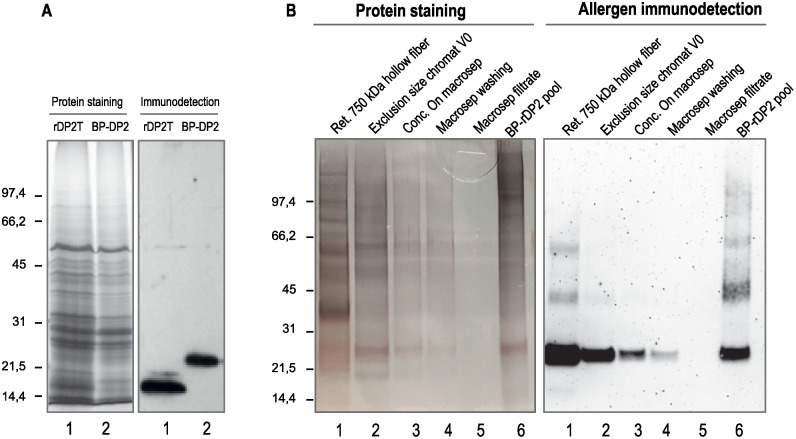
Expression and purification of BP-DP2. Protein extracts from leaves producing rDP2T (panel A, lane 1) or BP-rDP2 (Panel A, lane 2) were separated by SDS-PAGE and stained in gel or immunodetected par immunserum specific of Der p 2. BP-DP2 were then purified par different runs of filtration and chromatography on Hollow fiber (panel B, lane 1), exclusion size chromatography (lane 2) and macrosep (lane3). The final pool of BP is illustrated panel B, Lane 6.

Based on a detailed analysis of viral envelope proteins previously shown to induce VLP formation, we designed a Type I membrane protein carrier having the capacity to induce budding of the plasma membrane and bioparticle formation. We successfully expressed this carrier fused at the C-terminal end of Der p 2 allergen to produce bioparticles presenting this allergen at their surface (Fig 1). The electrophoretic mobility of the carrier fused to Der p 2 was compared to the electrophoretic mobility of rDer p 2 by immunoblotting. The difference in electrophoretic migration observed corresponded to the expected size difference between the soluble protein and Derp 2 fused to the carrier (Fig 5A).

**Fig 6:**
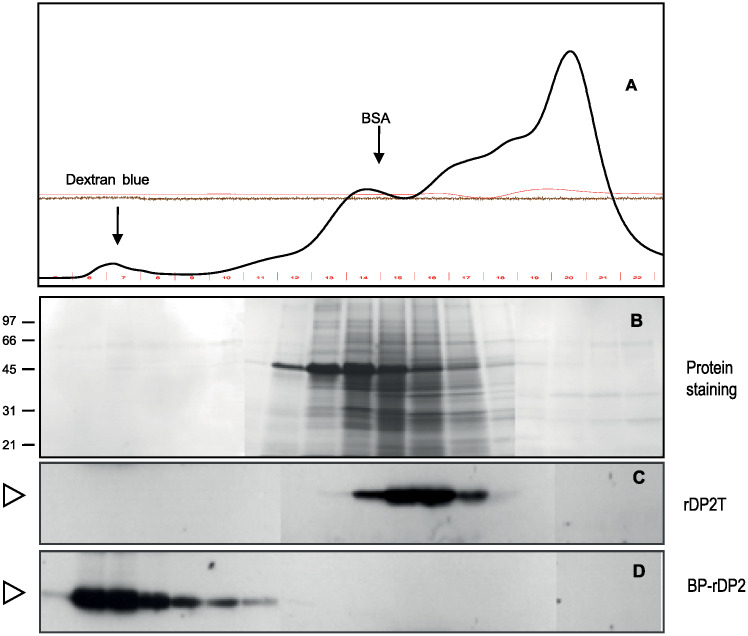
Purification and characterization of by steric exclusion chromatography. Protein extracts from leaves producing rDP2T (Panel C) or BP-rDP2 (Panel D) were separated by chromatography on a calibrated S-500 / HR column. The total soluble protein content of each fraction was evaluated by spectrometry (panel A) and protein staining with Coomassie Blue after separation by SDS-PAGE (panel B). The allergen content of the elution fractions was revealed by immunodetection using anti-DP immunserum.

Bioparticles presenting Der p 2 at their surface (BP-Der p 2) were purified from crude protein extracts using a two-step procedure. The first purification step is based on tangential filtration using a 750kDa hollow fiber (Fig 6B), then the bioparticles were separated from the contaminants still present using size exclusion chromatography. The BP were then sterilized and concentrated.

**Fig 7:**
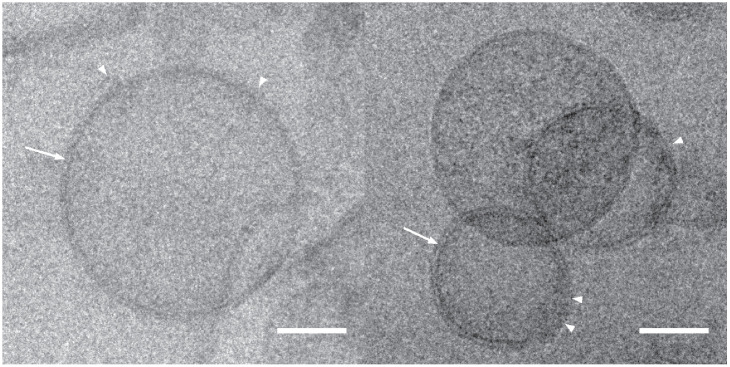
Observation of bioparticles in Cryo-EM. Bioparticles are spherical proteolipidic structures of about 150 nm. The presence of the lipidic scaffold of the supramolecular structures were confirmed since lipid leaflets were resolved (arrows). Presence of hairy structures at the surface of the supramolecular structures Presence of hairy structures at the surface of the supramolecular structures is highlighted by arrow-heads. Scale bars are 50 nm.

**Fig 8:**
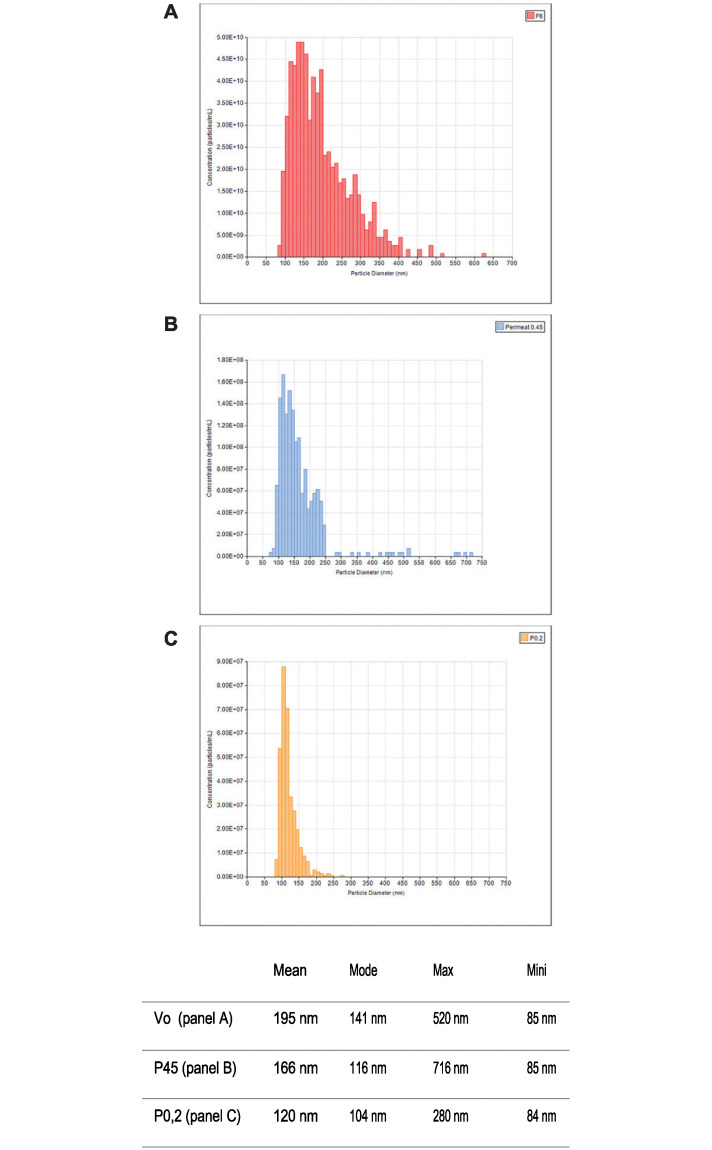
Comparative evaluation of tunable resistive pulse sensing of size distribution of BP-DP2 after purification (Panel A), filtration on 0.45 mm (Panel B) and 0.2 mm (Panel C). The graph represent averages of triplicate measurements.

The ability of the hybrid construct to assemble into supramolecular particles was fully validated by elution profiles using size exclusion chromatography on Sephacryl S-500HR. When partially purified extracts from leaves were loaded on a calibrated size exclusion matrix, supramolecular protein structures were excluded from the matrix and eluted in the fractions close to the solvent front as was Dextran Blue (Mw 2 000 000). Presence of the target allergen in the eluted supramolecular structures was confirmed by western blotting (Fig 6D). In addition, the pooled fractions containing the supramolecular structures were concentrated (Fig 5B, lanes 6) and their content examined under cryo-transmission electron microscopy (Fig 7) and through Tunable Resistive pulse sensing (Fig 8). Cryo-electron microscopy confirmed the presence of the lipidic scaffold of the supramolecular structures since lipid leaflets were resolved (Fig 7, arrows). Presence of hairy structures at the surface of the supramolecular structures are thought to represent the allergen (Fig 7, arrow-heads).

It was confirmed that in all fractions collected in the elution front that showed immunoreactive content (Der p 2), the supramolecular structures were present as bioparticles. Predictably, soluble rDer p 2 allergen migrated with the bulk of soluble proteins upon chromatography (Fig 6C).

### Regulation of allergic airways disease by rDer p 2 and BP-Der p 2

The ability of purified rDer p 2 or BP-Der p 2 to induce Th2 adaptive immunity was next examined in a murine model of allergic airways disease in which mice were sensitized via the intraperitoneal route with either Imject alum alone (as a control) or in the presence of rDer p 2 or HDM extract. Other mice were exposed similarly to BP-Der p 2, either alone or in the presence of Imject alum.

**Fig 9:**
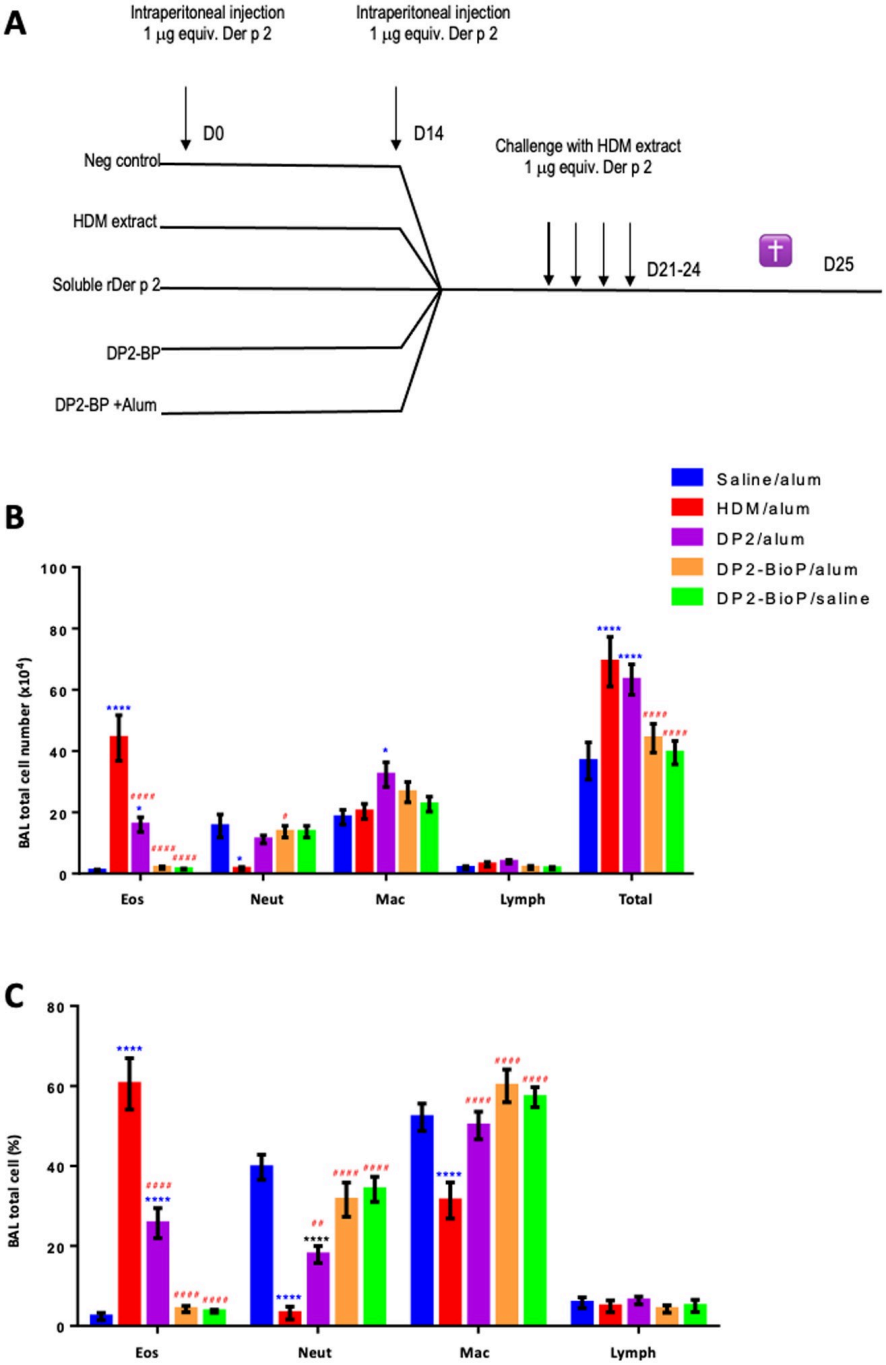
BALF eosinophil influx is increased by purified DerP2, but not by bioparticle-DerP2. Wild-type BALB/c mice were sensitized as described in methods and challenged with HDM extract after which BAL fluid was harvested and inflammatory cells quantified by differential counting (panel A). (B) Absolute number of BALF cells and (C) frequency of different BALF populations are shown. Data are from the combination of two independent experiments with a total of 7–8 mice per group and are presented as mean ± SEM. One-way ANOVA, Dunnett’s post hoc test comparing the mean rank of each column with the mean rand of saline/alum (* p < 0.05, **** p < 0.0001) or HDM/alum (## p < 0.01, #### p < 0.001).

**Fig 10:**
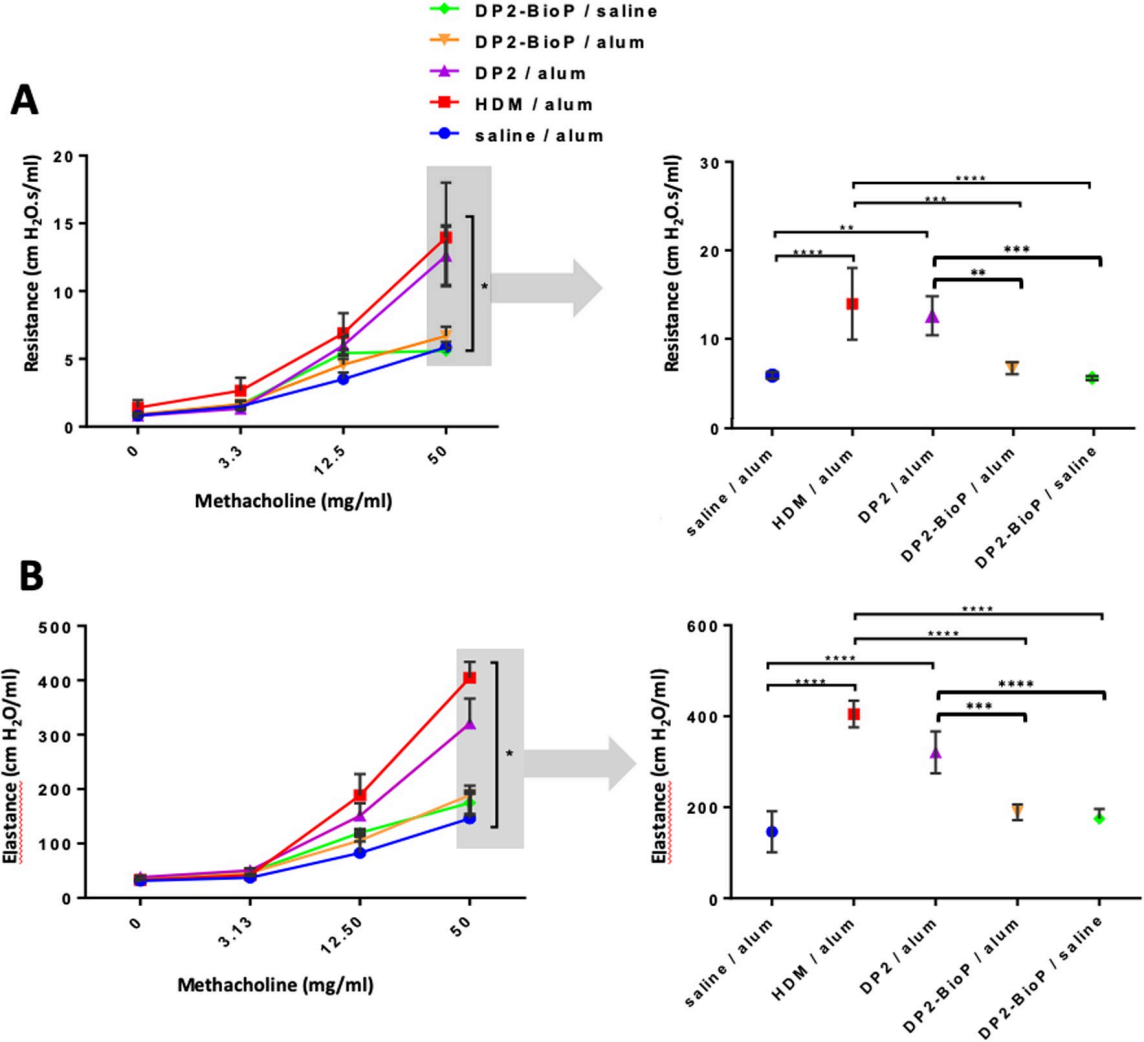
AHR is increased by purified DerP2, but not by bioparticle-DerP2. Wild-type BALB/c mice were sensitized as described in methods and challenged with HDM extract. Lung function was assessed with the flexiVent small animal ventilator and is presented as (A) lung resistance: dose response (left) and 50mg/ml dose (right) and (B) lung elastance: dose response (left) and 50mg/ml dose (right). Data are from one experiment representative of two using 4 mice per group. Data are presented as mean ± SEM. One-way ANOVA, Tukey’s post hoc test (* ≤ 0.05, ** ≤ 0.01, *** p ≤ 0.001 ***p ≤ 0.0001).

As illustrated in Fig 9A, after a 14-day rest, all mice were challenged with HDM extract after which several outcomes related to Th2 adaptive immunity were assessed—differential cell counts in the BAL fluid, induction of airway hyperresponsiveness, and levels of Der p 2 specific IgG. Following HDM challenge, total cells and eosinophils in the BAL fluid were highest in mice sensitized to either HDM or rDer p 2, whereas eosinophils were not recruited into the airways in mice sensitized with BP-Der p 2 (Fig 9). Mice sensitized with BP-Der p 2 (either alone or with Imject alum) showed cell population types similar to those in the control group. No significant difference was observed in the two BP-Der p 2 groups compared to the negative control while mice sensitized with purified rDer p 2 showed higher eosinophil counts, although these were lower compared to mice sensitized with HDM extract. Consistent with the eosinophilic inflammatory response in mice sensitized with either rDer p 2 or HDM extract, following HDM challenge, AHR was present in mice sensitized with either HDM or rDer p 2, as demonstrated by significantly greater total lung resistance (Fig 10A) and elastance (Fig 10B). In contrast, AHR was absent in mice sensitized with BP-Der p 2. Total lung resistance and elastance did not differ in these mice from those injected with control Imject alum.

**Fig 11:**
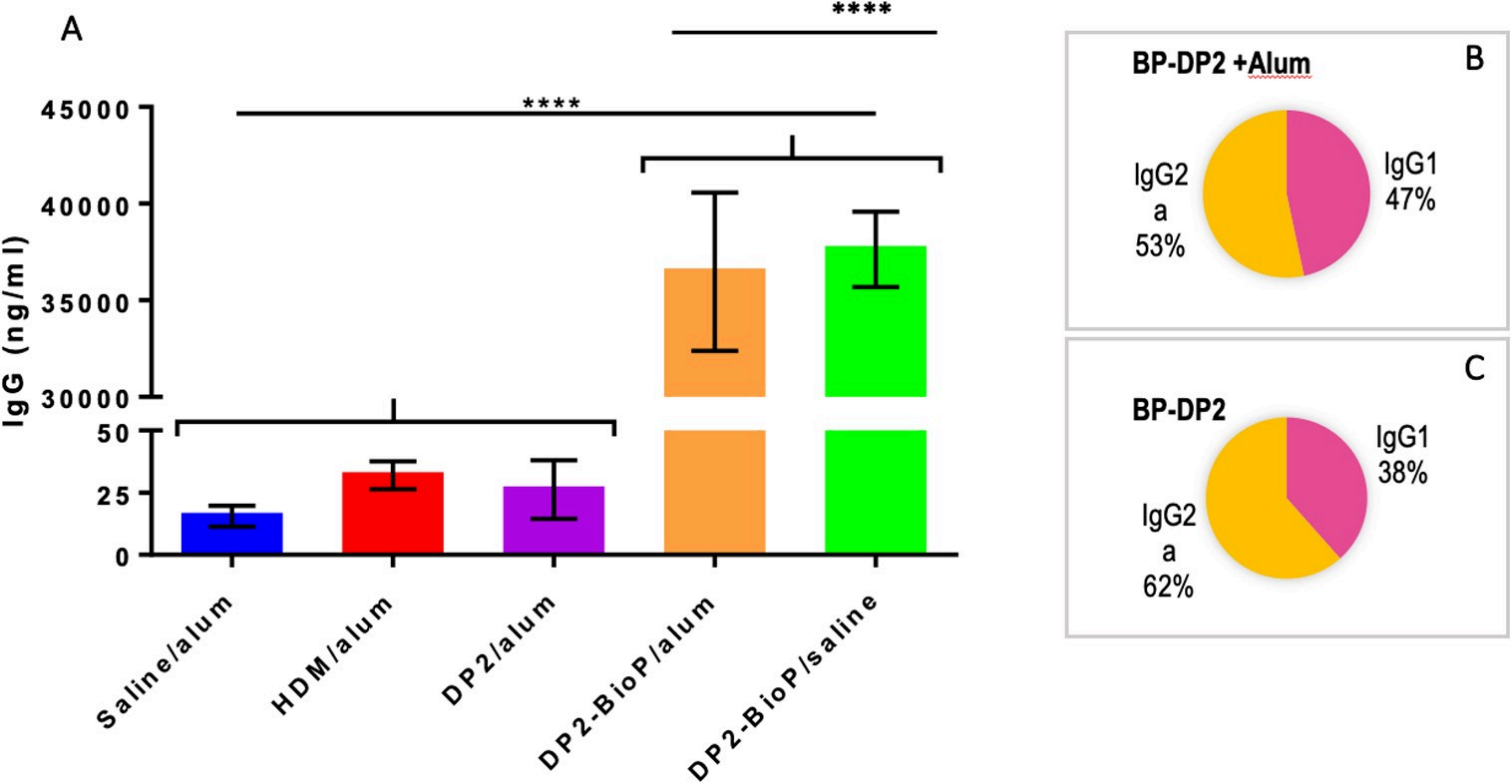
Der p2 specific IgG is dramatically elevated in mice sensitized with bioparticle-DerP2. Wild-type BALB/c mice were sensitized as described in methods and challenged with HDM extract. After sacrifice, serum levels of total Der P2-specific IgG were quantified. Data are from one experiment representative of two using 4 mice per group. One-way ANOVA, Tukey’s post hoc test (****p ≤ 0.0001). The ratio IgG2a/IgG1, was estimated after immunization with BP-DP2 in presence (Panel B) or in absence (Panel C). The results illustrate that the adjuvant are not necessary for effective immunization with BP.

Finally, we examined serum levels of Der p 2-specific IgG in mice sensitized with HDM, rDer p 2, or BP-Der p 2. While both HDM- and rDer p 2-sensitized mice had modest increases in Der p 2 specific IgG, levels of Der p 2 specific IgG were dramatically increased, over three orders of magnitude, in mice sensitized with BP-Der p 2. Allergens exposed on eBPs induced a very strong IgG response consisting predominantly of IgG2a. Indeed, the IgG2a / IgG1 ratio was very clearly in favor of the Th1 response, and results indicate that Th2-skewing is absent upon intraperitoneal (IP) exposure to BP-Der p 2, and that it can even override Th2 skewing by alum. (Fig 11). Altogether, these data provide evidence that Th2 adaptive immunity and allergic airways disease were efficiently induced in mice exposed to HDM or rDer p 2. However, delivery of BP-Der p 2 modulates this response, enhancing IgG production without skewing toward Th2 adaptive immunity associated with allergic asthma.

### Basophil activation

**Fig 12:**
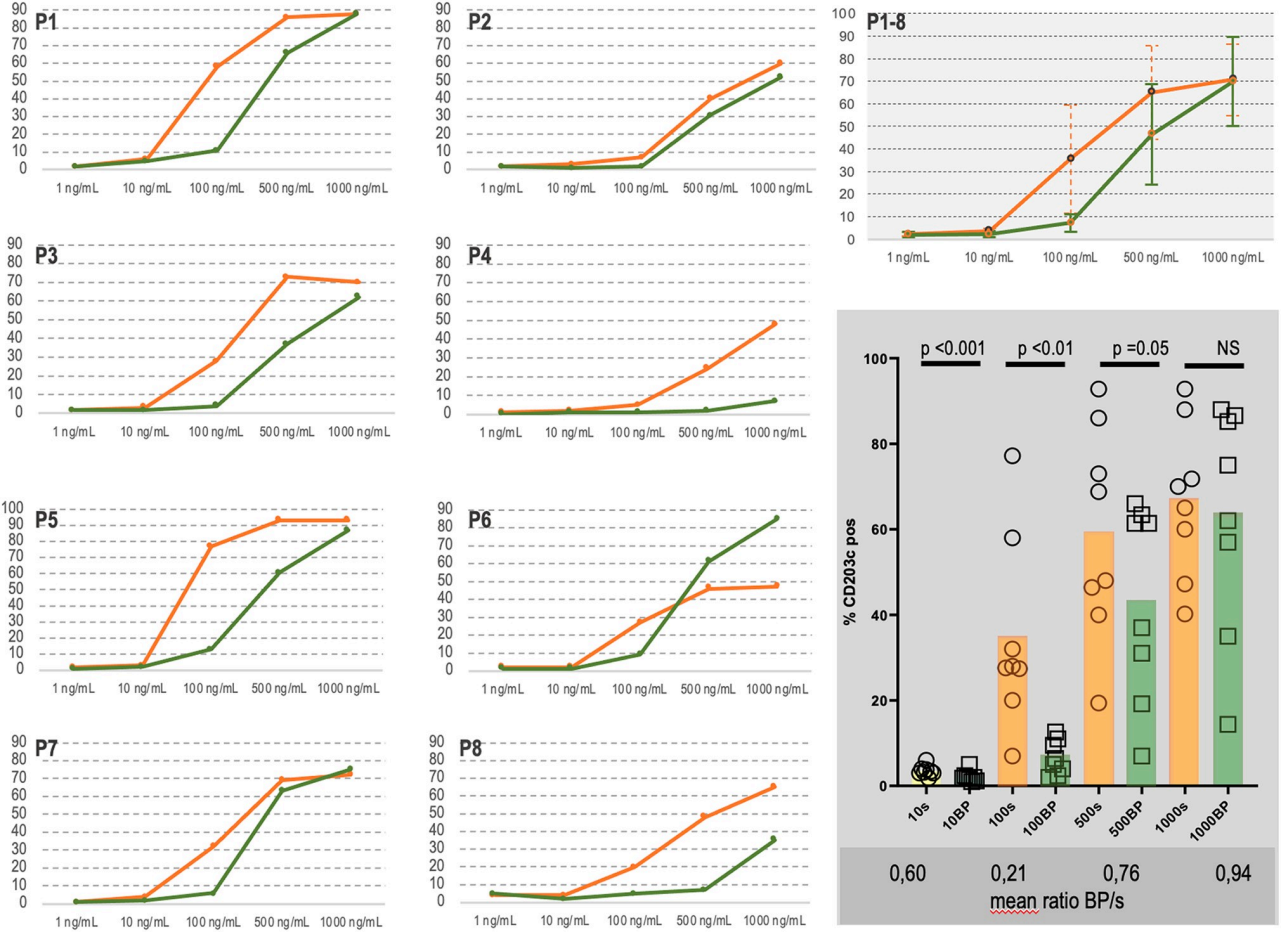
Influence of treatment of basophils with BP-Der p 2 (BP) or soluble Der p 2 (s) on CD203c upregulation at different concentrations (10,100, 500 or 1000 mg/ mL). The reduced activity was observed in all 8 HDM-allergic donors (P1-P8).

We next assessed the anaphylactic potential of the BP-Der p 2 compared to soluble Der p 2 in human basophils. To assess the allergenicity of BP-Der p 2, a basophil activation test was performed with cells from 8 HDM-allergic donors. Basophils were stimulated with titrated doses of Der p 2 or BP-Der p 2. We compared equal concentrations of rDer p 2 added to the cells, that is, amounts of BP-Der p 2 were normalized to Der p 2 content. Whereas soluble rDer p 2 (at 100 ng/ml) induced significant upregulation of CD203c, a measure of basophil activation, BP-Der p 2 did not (Fig 12).

## Discussion

### Transient expression of soluble Der p 2: Synthesis and maturation

It has been demonstrated repeatedly that plant cells, as many eukaryotic cells, can be used as host for the production of complex structural and bioactive proteins. Protein types ranging from small cytokines (interferon γ, IL-2) to the complex assemblies of IgMs or secretory IgAs have all been produced in plants, either with the use of true genetic transformation (stable integration of target DNA) or through different approaches of transient expression [56] (reviewed in [57]); [58–60]. As was shown for other eukaryotic expression systems, it has been demonstrated that molecular engineering can be used in plants for the control of protein trafficking, maturation, folding and most other post-translational modifications [61–64]. Plants are now emerging as a true recombinant platform. Some of the plant-based platforms have now reached industrial scale of operations. They equal and often surpass other recombinant platforms in terms of quality, and offer significant advantages over other platforms, in terms of cost efficiency, surge capacity, and even safety [65, 66].

Allergens make no exception. It has been shown that plants can produce mite allergens through stable genetic transformation [36]. In that study, the plant-made recombinant allergens were indistinguishable for their natural counterparts. They were correctly folded, fully glycosylated, and their physico-chemical properties were identical to the naturally sourced allergens. However, a long period of time was necessary for generating stable transgenic plants and the product yields were often very low.

In contrast, we show here that transient expression in *Nicotiana benthamiana* allows high-yield production of recombinant allergens. Interestingly, although high yield is observed, the protein maturation machinery is not saturated, with a perfect cleavage of signal peptide, and a folding of rDer p2 similar to the one of nDer p 2, including disulfide bridge formation. This is consistent with previous results illustrating that plant cells have a very sophisticated protein maturation machinery that allows the production of complex eukaryotic proteins [57, 67, 68].

### Design and production of allergen bioparticles

We report here the design and production of true allergen bioparticles, i.e. allergen-bearing bioparticles that are wholly synthesized and assembled by a host cell. These must be differentiated from other types of allergen bioparticles that are either *in vitro* assembled from synthetic biomaterials or nanoparticles that consist of *in vitro* assembled mixes of non-biomolecular and bio-molecular components.

VLPs (virus-like particles) are one type of bioparticle. Their components are of viral origin but they lack the elements that make viruses infectious. They occur naturally or can be produced by co-expression of the essential viral components in a host system. They generally have the structural characteristics of the viruses from which their components originate. They have been produced from a wide variety of virus families and in multiple cell culture systems. However, not all viruses have been produced as VLPs. For some (e.g., Ebola), it appears that the number and complexity of components required for assembly present too much of a challenge [69]. For others, the current knowledge required on the essential elements and assembly steps remains too scarce to be used in the design and production of VLPs [70].

VLPs contain repetitive displays of viral conformational epitopes that can elicit strong T cell and B cell immune responses, which make them high potency vaccines. VLPs can be made from self-assembling capsid proteins, or from transmembrane viral proteins that must be processed by the host cell for assembly. In general, VLPs have been used to present major viral surface immunogens to act as vaccines [20]; however, as these surface immunogens are often also part of the viral (or VLP) structure, in VLPs, structure is linked to immunogenicity. Said differently, one cannot produce a VLP that is not immunogenic and as such, VLPs cannot be used to present other immunogens, without creating an anti-viral immune response. The corollary to this is that the use of VLPs as anti-viral vaccines remains limited to those viruses for which sufficient knowledge on the structural composition and assembly is available, and to those where natural assembly principles can be reproduced in foreign living cells.

In this study, our goal was to produce bioparticles that could present major allergens. The objective was to exploit the immunomodulatory potential of enveloped bioparticles, while avoiding the triggering of an immune response to viral surface epitopes and the limitations posed by the structural mechanics of natural virus assembly.

Starting from the hybrid bioparticle concept described by Compans and collaborators [30], we designed a protein construct in which GCN4 was used both for presentation of allergens at the surface of the bioparticle, and for the polymerization of Der p 2 monomeric constructs. Taken together, these results suggest that as for natural viral transmembrane proteins, chimeric allergen polymers can assemble at the surface of the plasma membrane and induce the curvature and budding out of lipid/protein assemblies. It has been demonstrated herein that chimeric allergen polymers accumulate as bioparticles and, as first demonstrated by Compans and colleagues [30], for core viral proteins, that assembly of enveloped bioparticles is not dependent on either the use of viral polymerization domains, or the presence of viral matrix proteins.

The fusion protein used in the present study is made of four different peptides; the allergen, here Der p 2, a coiled-coil trimerization domain based on GCN4 transcriptional activation factor from *Saccharomyces cerevisiae* [47], and a synthetic membrane anchoring domain consisting of a transmembrane segment and a cytosolic segment. When this fusion protein is expressed, after trimerization, intracellular transport and accumulation in specific domains of the plasma membrane, this membrane buds and form bioparticles. During its intracellular transport in the luminal part of the secretory pathway the allergen fused at the N-terminal end of the membrane carrier is folded and matured. After budding and bioparticle formation, allergens and oligomerization sequences are finally exposed outside the bioparticle.

Compared to the complexity of previous attempts to produce allergen-bearing VLPs derived from the bacteriophage Qß [71–77], one of the great advantages of allergen-bearing BPs produced in this study, is that they are completely assembled within the host cell and secreted under their mature form between the plasmalemma and the plant cell wall.

Another advantage of allergen-bioparticles produced in this study, compared to allergen- QßVLPs developed by others for allergen immunotherapy, is the complete absence of virus surface protein. When allergen-Qß VLPs are used for immunotherapy, virus proteins are co-injected with the allergen of interest, so that virus vaccination could occur as a side effect of immunotherapy against allergen as described in Cornelius et al. [78]. This side effect was also clearly demonstrated for QßVLPs made of a peptide sequence from the HDM allergen Der p 1 fused to the coat protein from the bacteriophage Qß. When this Der p 1-QßVLP was injected in humans, the antibody response against the carrier Qß was higher than the response against Der p 1 [71].

Calculations (not shown) based on the concentration of particles (TRSP) and the content in Der p 2 (as estimated by calibrated western blotting) suggest that each BP contains between three to four thousand repeats of Der p 2 molecule on its surface and that the density of allergens on the surface of BP-Der p 2 is highly reproducible from batch to batch. This is in contrast with Qß bioparticles where the 180 constitutive Qß monomers are either free or covalently bound to 0, 1, 2, 3 or more allergen molecules [71]. Surfaces containing highly repetitive antigens, along with the size of the bioparticle facilitate interaction with APCs and their transport to tissue-draining lymphoid organs.

Compared to the complexity of previous attempts at producing allergen-bearing VLPs derived from the bacteriophage Qß [72] one of the great advantages of plant-made allergen-bearing BPs is that they are completely assembled *in planta* and secreted under mature form between the plasmalemma and the cell wall of plant cells. Production of allergen-BP is thus simple, fast and inexpensive.

### Immunomodulation potency of allergen bioparticles

Intraperitoneal (IP) exposure of mice to alum-adsorbed allergens is commonly used to sensitize mice to allergens to generate a Th2 adaptive immune response. Subsequent delivery of allergen to the lungs induces many of the typical hallmarks of the asthmatic lung: increased eosinophil recruitment in the lungs and airways as well as AHR [79]. Also, subcutaneous exposure to alum-adsorbed allergens has been reported to induce Th2-type sensitization [80]. Here we confirmed that IP exposure to alum-adsorbed HDM extract or alum-adsorbed rDer p 2 indeed induced AHR and eosinophil accumulation into the airways, upon subsequent challenge with HDM, although the amplitude of response to rDer p 2 was lower than that of HDM. Interestingly, BP-Der p 2 did not induce eosinophil recruitment or AHR. Indeed, mice exposed to control alum or BP-Der p 2 showed similar populations of cells in BAL and lacked AHR (Figs 10 and 11). Furhtermore, these data also demonstrate that alum was not of any benefit when the mice were sensitized with BP-Der p 2. Moreover, BP-Der p 2 contributed to balance the Th1/Th2 response even if administered in the presence of alum. This indicates that Th2-skewing is absent upon IP exposure to BP-Der p 2, and that it can even override Th2 skewing by alum. Further experiments are ongoing to characterize the immune-skewing properties of the allergen-BP in more detail. One of the aspects that deserves specific attention is the reported anti-inflammatory potency of glucosylceramides, which are a major component of the plant BP [81].

IP exposure to Der p 2 in the context of alum, either as HDM or purified Der p 2, resulted in only weak Der p 2-specific IgG responses, whereas BP-Der p 2 induced a very strong IgG response consisting predominantly of IgG2a. Indeed, the IgG2a / IgG1 ratio was very clearly in favor of the Th1 response in the absence of alum. These data contrast with the results obtained by Soongrung et al. [77] who demonstrated that, despite a very strong increase in IgG in the presence of VLP-Der p 2, the presence of phage does not modify the IgG2A / IgG1 ratio always in favor of IgG1.

Altogether, these results demonstrate that plant-derived allergen-bioparticles may be a very potent alternative to alum-based vaccines for allergen immunotherapy (AIT): strong allergen-specific IgG responses in the absence of pro-allergenic Th2 skewing. In vitro experiments with human monocyte-derived dendritic cells are currently being performed to establish the immune-skewing potential of BP-Der p 2. Preliminary results provide support for strong induction of the anti-inflammatory cytokine IL-10. Recently, regulatory authorities have questioned whether repeated exposure to alum, as is the case for many currently marketed AIT products, is desirable, in particular in children [8]. Moreover, adjuvants like aluminum hydroxide, if not formulated in an optimal ratio can impact the release of the antigens and ultimately influence the immunogenicity of the recipient [82, 83]. Finding alternatives for alum therefore is important and our plant-based bioparticles may provide an answer.

From a safety perspective, intuitively it appears that bioparticles expressing many copies of an allergen on their surface would be a risk to induce very efficient cross-linking of IgE on the surface of effector cells, i.e. basophils and mast cells. The contrary was the case: soluble counterpart, rDer p 2 present on the surface of BPs show a very limited potential to activate the basophil degranulation of patients allergic to the same allergen. As illustrate by the accumulation of the CD203c marker. Our results are in line with previous reports and particularly with an earlier publication on VLP presenting the major cat allergen Fel d 1 on its surface [73, 84, 85].

After a first sensitization, basophil degranulation occurs upon contact with the allergen. Recognition of this allergen by IgE carried by the FcεRI receptor at the surface of basophils (or mast cells) leads to activation of these cells, which is followed by release of granule products, including histamine. This activation requires that the allergen be multivalent or at least bivalent, allowing to link together two adjacent IgEs. Several approaches have been taken to improve the safety of allergen specific immunotherapy such a using hypoallergenic allergens or allergen peptides derivatives showing reduced IgE reactivity.

Here, we have shown that rDer p 2 presented at the surface of bioparticles was far less allergenic in BAT essay than its soluble counterpart. We have hypothesized that interaction of BP-Der p 2 with IgE bound to activated basophils is greatly reduced compared to rDer p 2 for simple physical reasons. Indeed, according to our results, BP-Der p 2 are about hundred times smaller than human basophils, the phenomenon called "bridging" or aggregation of IgEs by the allergen is thus strongly limited due to steric hindrance when allergens are presented on the surface of a BP. Altogether these results are predictive of a high safety potential for these allergen-BP for allergen immunotherapy.

## Conclusion

Replacement of crude allergen extracts by selected allergens currently represents a major goal for improvement of allergen immunotherapy. The development of molecularly-defined recombinant allergens would facilitate both standardization and enhance batch-to-batch reproducibility as well as treatment specificity.

This new expression platform using transient expression in *Nicotiana benthamiana* is used not only the production of soluble natural-like allergens but also to develop an immunostimulatory allergen presentation system that triggers a strong Th1/Treg-biased response. The delivery of antigens to the immune system as a particle form has been shown to induce a stronger antigen-specific immune response than soluble antigens [86, 87]. The size of the BP is ideal for antigen presenting cells (APCs) uptake and the presence of highly ordered repetitive structures on their surface provides a signal to initiate immune activation [88].

With a diameter of about 170nM, bioparticles presenting the allergen Der p 2 on their surface have the perfect size to be very efficiently taken up by dendritic cells [89, 20], and reach lymphoid organs by direct diffusion through the 200 nm pores of the lymphatic vessel walls, within a few hours after injection, to induce a potent immune response after immunization [90, 91, 70].

Moreover, in keeping with what has been shown for new generation of vaccines (Bachmann and Jennings 2010 [92]) our results obtained using BP-Der p 2 show a degree of allergenicity indistinguishable from placebo, while stimulating a strong IgG response.

Combining a new manufacturing technology and a new 3D allergen display technology, we have now established the capacity to produce tools bridging the gap between molecular allergology and clinical treatment.

## Supporting information

S1 Raw images (PDF)
