# Supplementary material for: Design, production and immunomodulatory potency of a novel allergen bioparticle
Source: PLoS One. 2020 Dec 1;15(12):e0242867. doi: 10.1371/journal.pone.0242867 (PMC7707610; doi:10.1371/journal.pone.0242867)
Supplement: S1 Raw images — (PDF) [file pone.0242867.s001.pdf]

Figure 2:

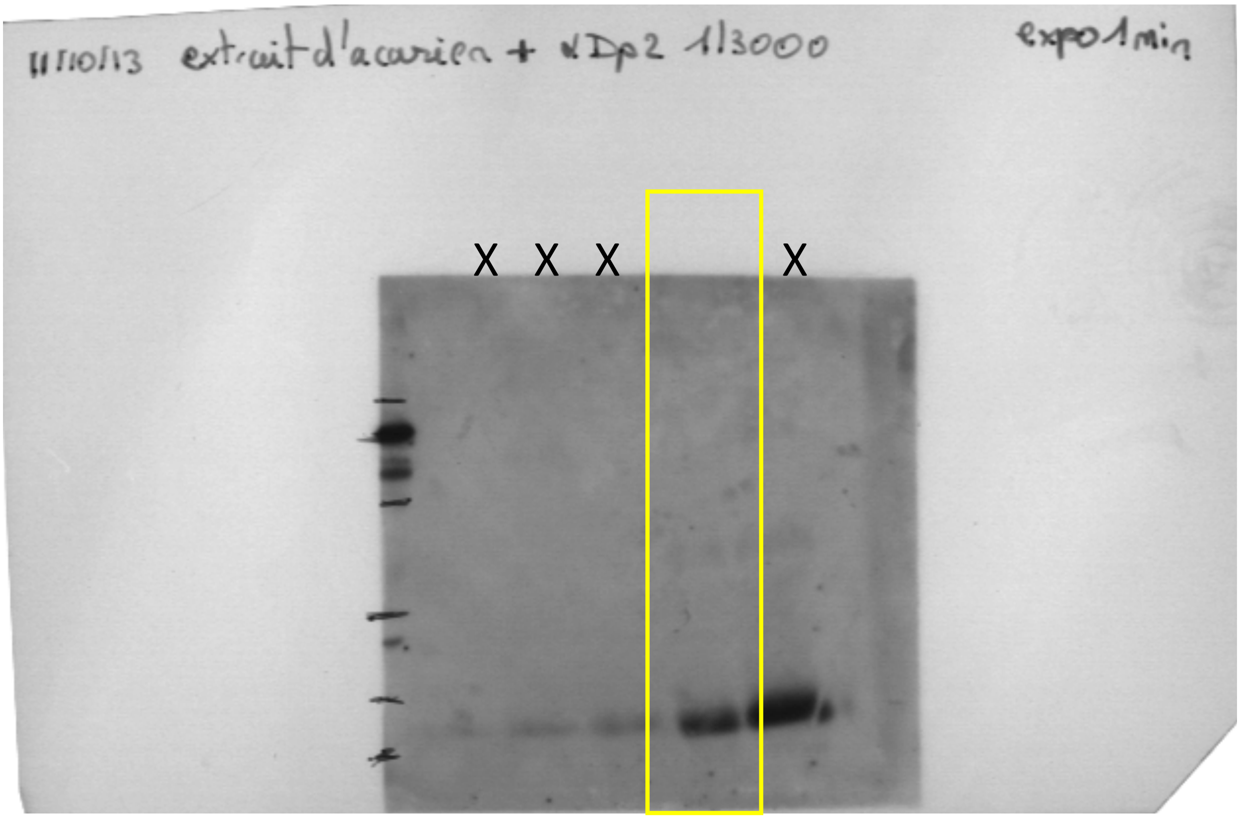
11/10/13 extrait d'acarien + αDp2 1/3000
expo 1 min
X X X X

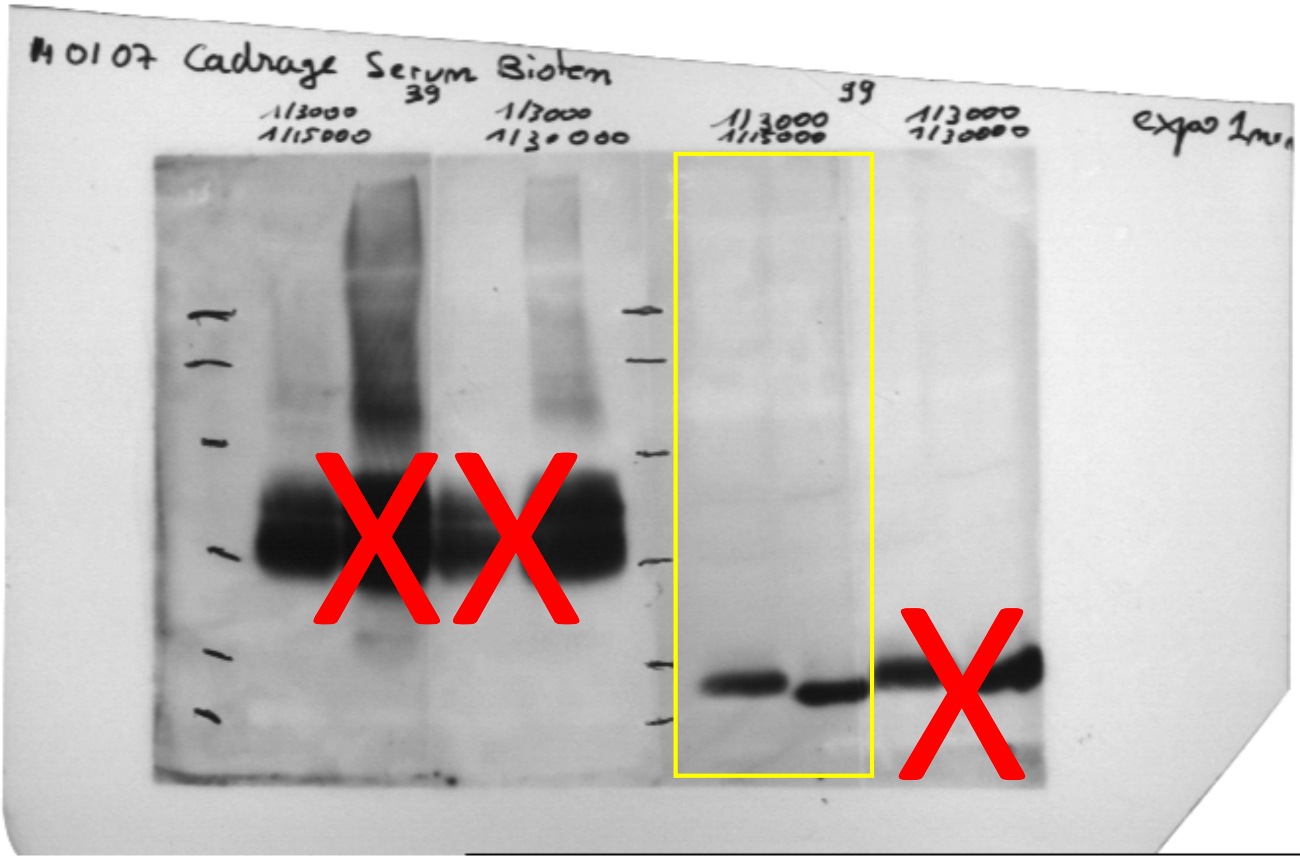
11 01 07 Cadrage Serum Bioten
39
1/3000
1/15000
1/3000
1/30000
39
1/3000
1/15000
1/3000
1/30000
expo 1 min

Figure 3:

We are sorry, having changed the image acquisition system, we no longer have access to the original image which made it possible to compose figure 3. However, this experiment has been carried out many times and I allow myself to provide you with the original images of the various purifications. You can observe that the purification process is very reproducible.

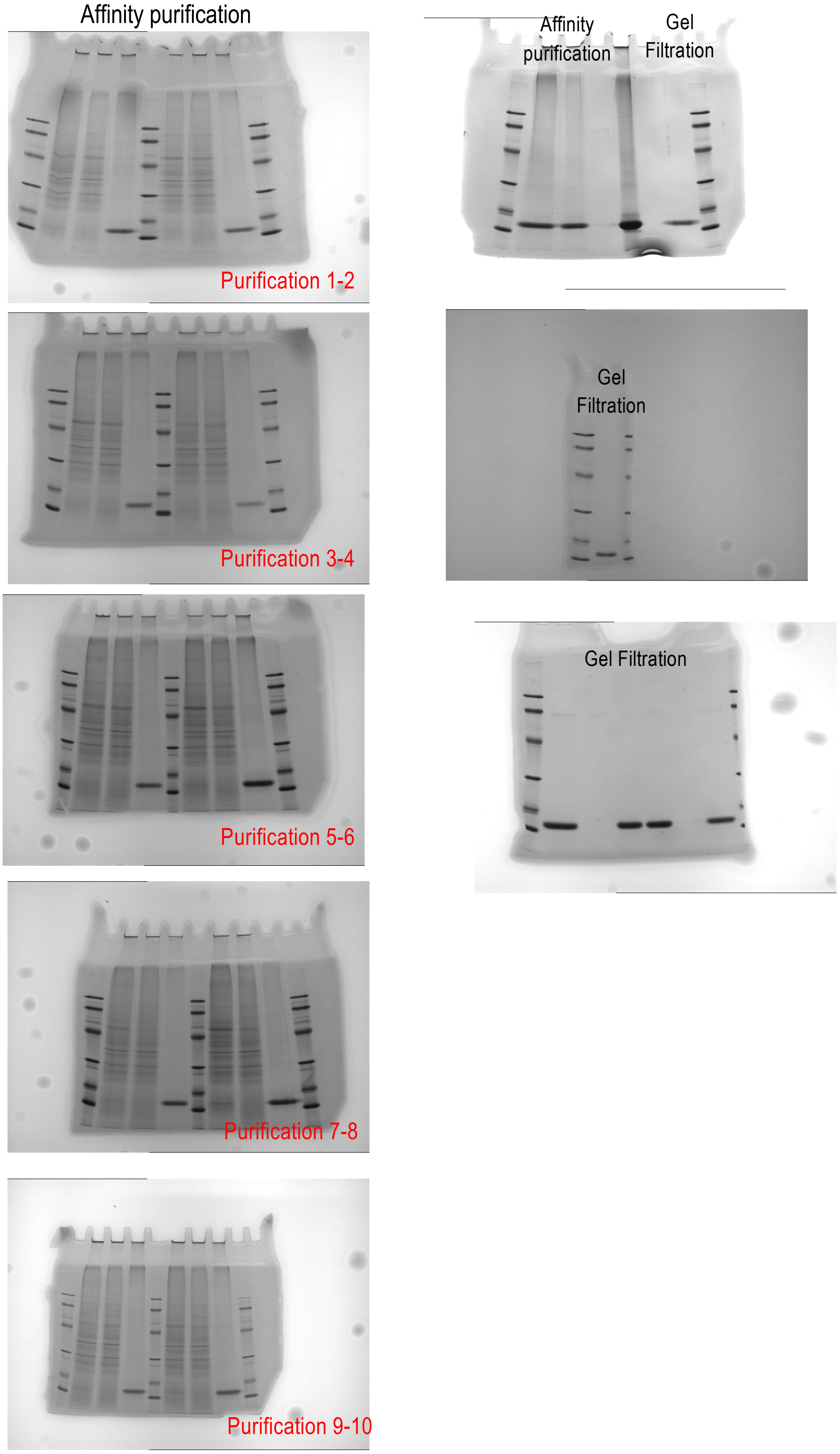

Figure 5 :

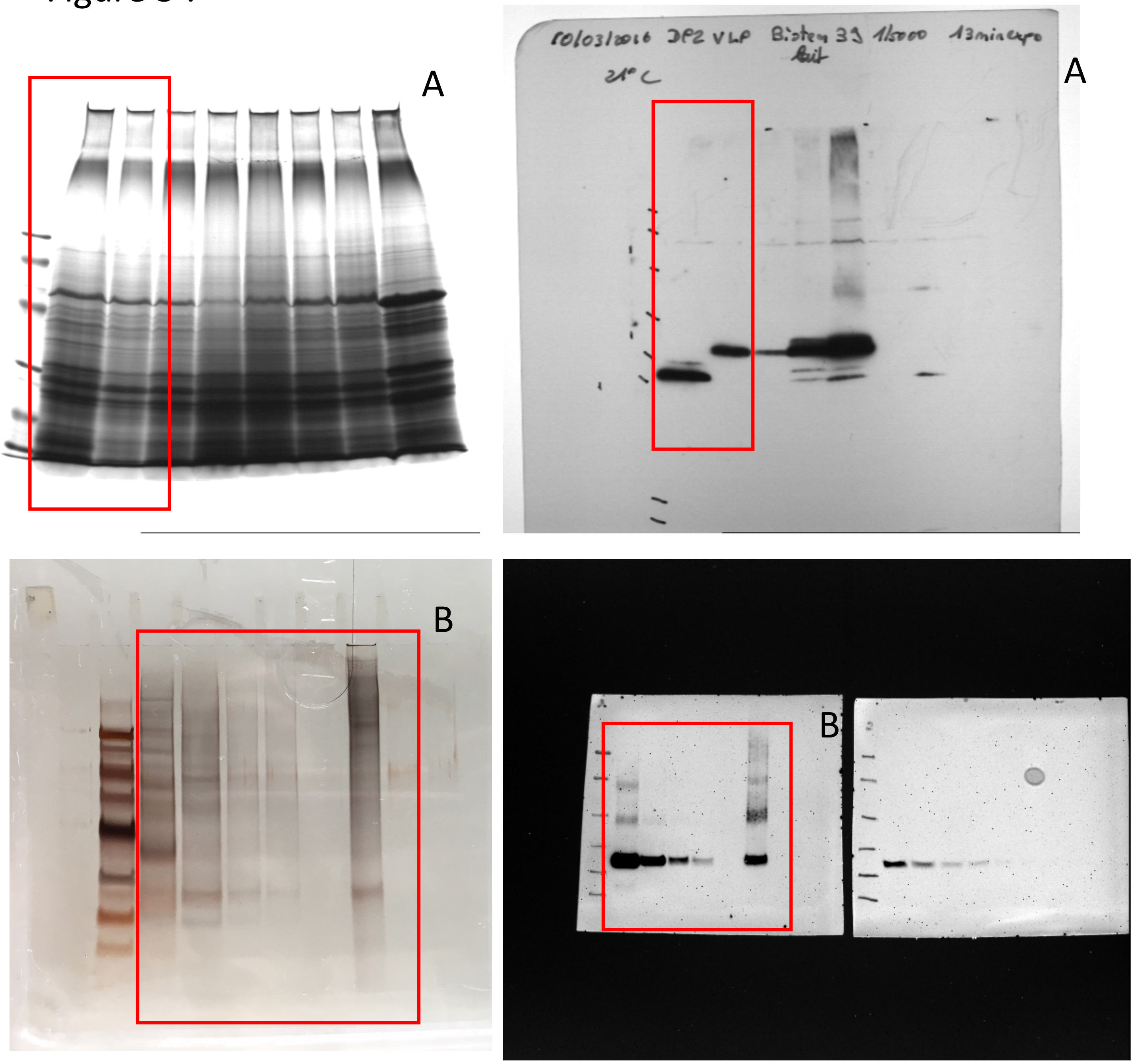

Figure 6 :

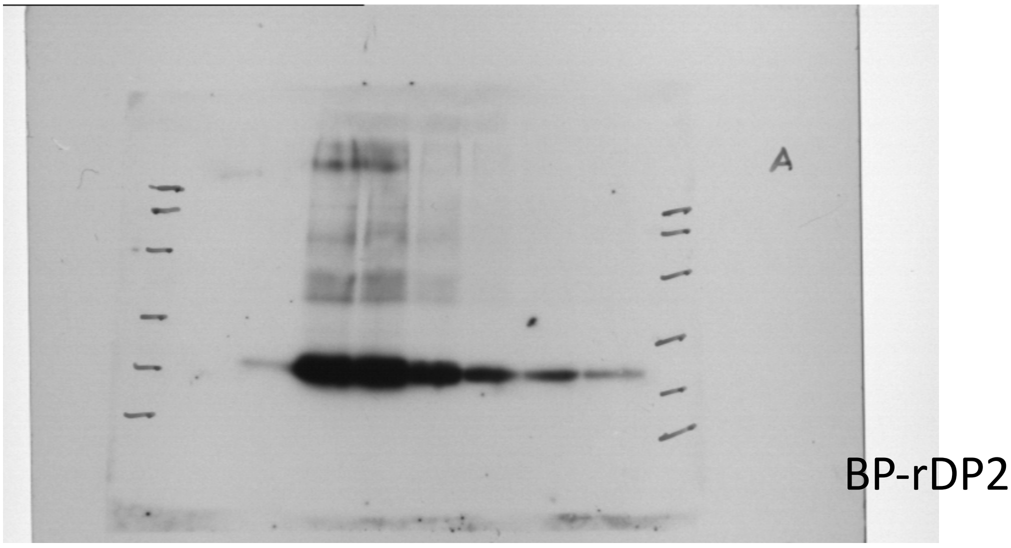

BP-rDP2

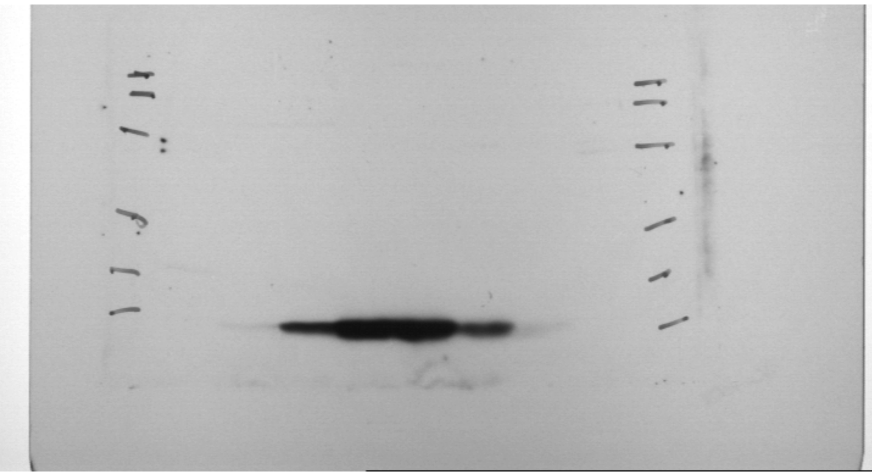

rDP2T
